# Response to Letter to the Editor: “Trends in Endocrinology Fellowship Recruitment: Reasons for Concern and Possible Interventions”

**DOI:** 10.1210/clinem/dgaa352

**Published:** 2020-06-02

**Authors:** Giulio R Romeo, Irl B Hirsch, Robert W Lash, Robert A Gabbay

**Affiliations:** 1 Joslin Diabetes Center, Harvard Medical School, Boston, Massachusetts; 2 Division of Endocrinology, Diabetes and Nutrition, University of Washington, Seattle, Washington; 3 Endocrine Society, Washington, District of Columbia

We appreciate the comments by Malek and Lamos (1) that raise several important issues pertaining to the professional development of clinical educators in endocrinology.

The letter highlights that the prevailing system used by many academic Institutions fails to adequately measure and value the essential role of clinical educators. Because criteria for academic promotion heavily rely on high-visibility publications, the modest interest by prominent endocrinological journals for innovative, teaching-oriented manuscripts indirectly stymies educators’ career advancement.

As outlined in our article, we agree with Malek and Lamos (1) that supporting the professional growth of committed clinical educators (and therefore their job satisfaction) is an essential conduit for attracting medical students and residents into endocrinology. We also concur with prompting endocrinology organizations to enhance the footprint of pedagogic research in their leading journals. For instance, an active flow of information in this space has never been as essential as during the current COVID-19 pandemic, which has imposed swift and creative transformations in curricula at all levels of training (2, 3). Studies that compare and contrast different models of medical education in the nearly unprecedented scenario of “social distancing” and telemedicine would be of interest to the general audience of endocrinologists, and deserve far-reaching platforms.

More broadly, the reappraisal of the clinical educator track—invoked for decades (4)—requires establishing metrics of achievement that extend beyond the publication record (5). Medical schools and affiliated institutions should grasp that a failure to retain clinical educators threatens to disrupt their core mission. In a single-center study, lack of professional development and institutional recognition for excellence in teaching are main reasons for early attrition (6), corroborating previous findings on a larger pool of medical school full-time faculty (7). Any attempt to increase the pipeline for new endocrinologists depends on exposure to motivated and effective clinical educators, who generally engage in teaching activities for reasons other than financial incentives (8). This point is exemplified by the increasing spontaneous participation of nonacademic endocrinologists to virtual teaching conferences, an interest that cannot be explained by economic reward.

In this context, endocrinological associations have the opportunity to champion medical education by promoting an expansion of the scope of their journals, as suggested by Malek and Lamos (1), by advocating for a stronger representation of physician educators in leadership roles, and by incorporating sessions on innovative educational approaches in the main agenda of national meetings. The latter aspect is particularly important as clinical educators’ exposure to formal training on adult learning techniques is often limited.

We believe this multitiered strategy would project an image of excitement, inclusiveness, and collegiality of our field that could boost the enthusiasm for entering a career in endocrinology.

## Additional Information

***Disclosure Summary:*** The authors have nothing to disclose.
